# Reconstruction of a neglected hyperextension-bicondylar tibial plateau fracture 9 months after original injury and review of the literature. What outcomes can be expected?

**DOI:** 10.1016/j.tcr.2023.100823

**Published:** 2023-03-14

**Authors:** Sophia M. Wakefield, Vasileios P. Giannoudis, Peter V. Giannoudis

**Affiliations:** a Academic Department of Trauma & Orthopaedics, School of Medicine, University of Leeds, Leeds, United Kingdom; b NIHR Leeds Biomedical Research Centre, Chapel Allerton Hospital, Leeds, United Kingdom

**Keywords:** Tibia, Plateau, Bicondylar, Fracture, Non-union, Management

## Abstract

Tibial plateau fractures range from simple to complex. Most complex injury types are managed surgically but for some, a decision is made to treat without surgery. We present a case that was managed non-operatively but due to failure of bone union, later required surgical intervention. We discuss the choice of management and potential risk factors influencing outcome.

## Introduction

Tibial plateau fractures are rare, accounting for 0.8 % of all fractures [1]. Different types exist with bony tibial disruptions observed in different planes with emergent gaps, depressions and displacements of bone surfaces. In most cases, there is no associated injury to the capsule, meniscus and/or ligaments of the knee [1].

Diagnosis is primarily made using X-ray, but CT scanning is essential to accurately delineate injury extent. The “four-column and nine-segment” classification stages hyperextension tibial plateau fractures (HTPFs) into four-levels based on injury mechanism, fracture morphology and additional injuries incurred [2]. Surgeons use this system to evaluate initial injury, plan management and predict prognosis, including the likelihood of associated soft-tissue involvement.

Displaced fractures are usually managed operatively, with the aim of restoring articular surface, correcting limb axis and fracture stabilisation, thereby facilitating early range of motion (ROM) and preserving injured cartilage [3]. Nonetheless, rarely, the fracture is not reconstructed due to patient comorbidities or a decision is made to allow fracture healing before considering a joint replacement, as some patients may have pre-existing degenerative changes.

Herein, we present a neglected tibial plateau fracture, initially managed non-operatively but later developed non-union associated with deformity and intrusively painful symptomatology.

## Case presentation

**Fig. 1:**
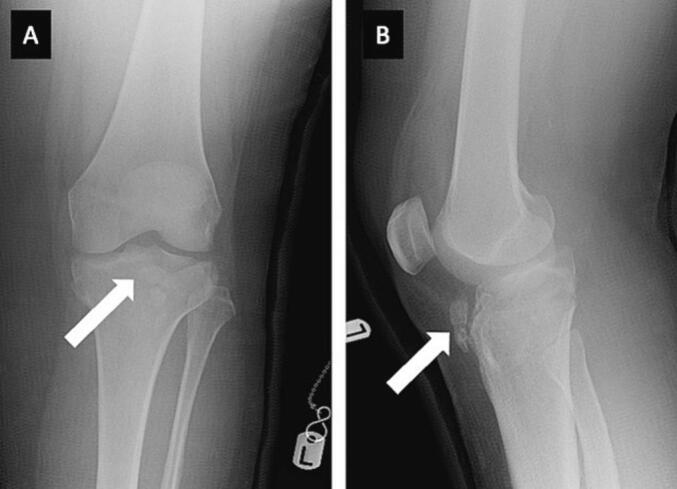
Anteroposterior (A) and lateral (B) radiographs of the left knee joint, obtained at injury presentation. These X-ray images demonstrated a hyperextension-bicondylar communited injury of the left tibial plateau (white arrows).

**Fig. 2:**
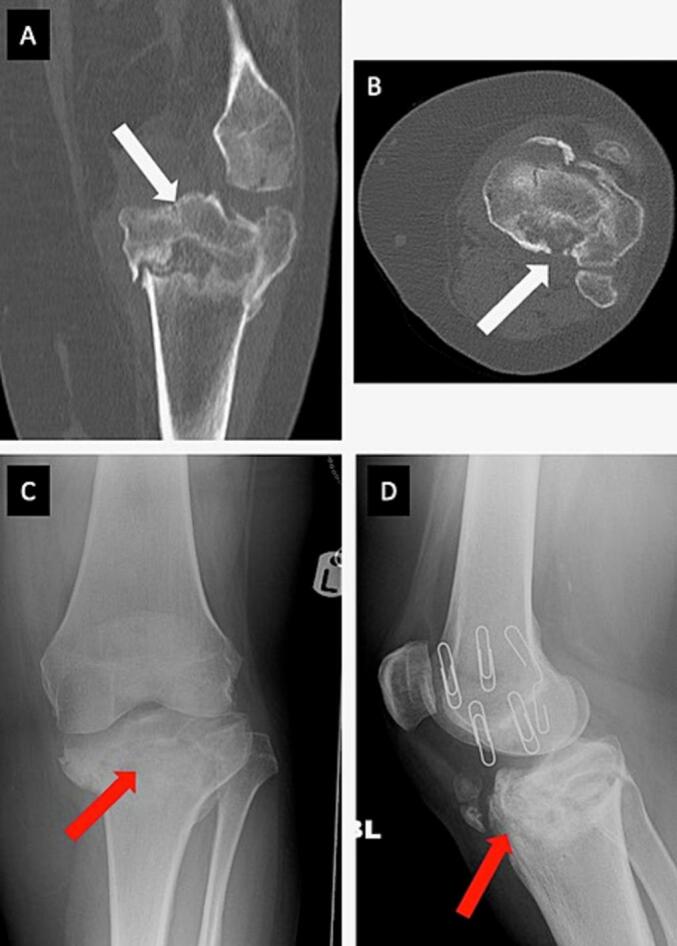
A & B) CT imaging of the left knee joint demonstrating fracture union of the left tibial plateau (white arrows); C & D) X-ray images obtained at initial presentation to our centre. These radiographs demonstrate (red arrows) a non-united bicondylar tibial plateau fracture in hyperextension and a varus position. (For interpretation of the references to colour in this figure legend, the reader is referred to the web version of this article.)

A female in her early 50s, who fell at ground-level, sustained a hyperextension-bicondylar communited left tibial plateau injury. At the time, alignment was deemed acceptable (Fig. 1), and she was managed non-operatively at her local hospital. Regular monthly follow-up X-rays were obtained, but at 6-months, as symptoms remained, a CT scan was performed, demonstrating delayed fracture union (Fig. 2). An exogen bone stimulator was prescribed to promote healing, with advice that a total knee replacement (TKR) may be warranted in the future.

She presented to our institution 9-months post-injury with ongoing, worsening daily pain (8/10 severity), especially on weight bearing (WB), which limited her ability to walk short distances, climb stairs and perform daily activities. She had undergone three previous surgeries for degenerative spine disease, with her spinal symptoms contributing to limited functional capacity and mobility aid reliance. She was morbidly obese (BMI 42.1), a longstanding smoker, and had a medical background of asthma, epilepsy and depression. Clinical examination revealed global tenderness on palpation around the knee joint, particularly over the medial aspect. She was neurovascularly intact. Blood results were unremarkable, except for low vitamin-D levels. X-rays demonstrated a non-united bicondylar tibial plateau fracture in a varus position, HTPF type hyperextension-bicondylar (Fig. 2).

**Fig. 3:**
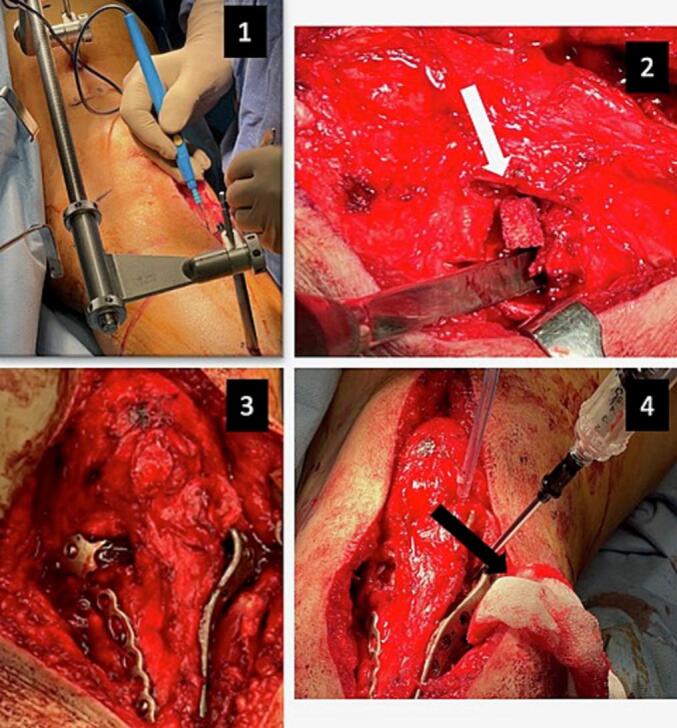
Intra-operative images demonstrating: 1) the application of a femoral distractor to correct the angular deformity and restore joint alignment, with a subsequent anterior knee midline incision performed; 2) the implantation of Tutobone (xenograft) (white arrow) to fill the gap and maintain reduction; 3) the application of bilateral plating on both medial and lateral sides of the plateau; 4) the filling of any remaining gaps with Hydroset bone substitute (black arrow).

Two potential treatment modalities were offered to the patient: surgical plateau reconstruction or a TKR. Although pre-injury function was suboptimal, she remained hopeful she would regain some mobility and independence. Reconstruction was therefore elected (Fig. 3). Preoperative planning included disimpaction/mobilisation of collapsed segments, correction of deformity and restoration of anatomical joint congruity. Under general anaesthetic, in a supine position, a femoral distractor was applied to correct angular deformity and restore mechanical axis and alignment, and a midline anterior knee incision was made to access both tibial plateaus. This approach was elected, accounting for the possibility the patient may eventually require a TKR. Using osteotomes under image intensifier control, the medial plane of the non-union was first identified, the depressed segment mobilised, and the varus and hyperextension malalignment corrected. A Tutobone (xenograft) wedge block was implanted to fill and support the gap created, thus maintaining reduction. Following this, the lateral plateau non-union was reduced through a lateral capsulotomy. Having anatomically corrected both fracture sites, bilateral plating was applied; Evos (Smith & Nephew, Watford, UK) and ALPS (Zimmer Biomet, Warsaw, Indiana, USA) proximal tibial standard plates were applied on the medial and lateral sides respectively. Any remaining gaps were filled with Hydroset bone substitute, and the wounds closed in layers.

**Fig. 4:**
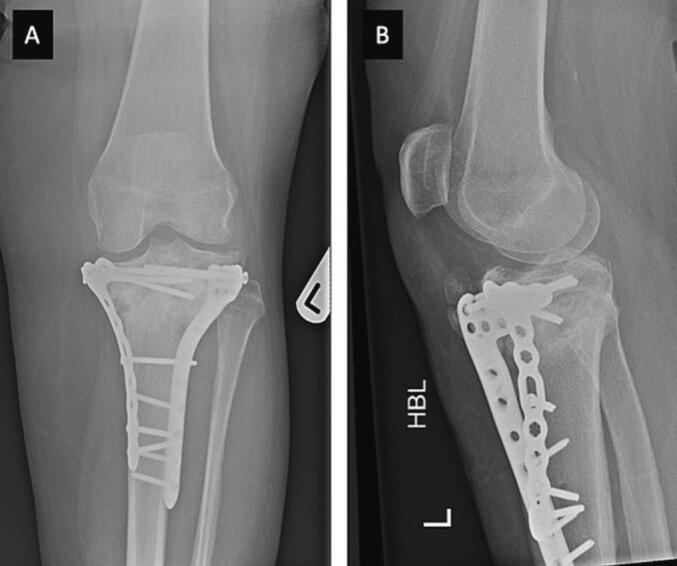
Post-operative anteroposterior (A) and lateral (B) radiographs at 12-months follow-up, demonstrating bilateral plating and healing of the fracture site.

Post-operatively, immobilisation using a knee brace, initially locked at 0–30 degrees of flexion, antibiotics (Flucloxacillin for 2 days) and thromboprophylaxis (Enoxaparin 40 mg for 8 weeks and anti-embolism stockings) treatments were adopted. Knee ROM was gradually increased every 2-weeks, reaching full, free ROM at 8-weeks. The patient progressed to partial WB at 12-weeks, after which she started full WB. Left knee radiographs were obtained at 2, 6 and 12-weeks and thereafter at 6 and 12 months (Fig. 4). At final follow-up, her EuroQual index [4] was 892. On examination, she had full knee extension with 100 degrees of flexion.

## Discussion

HTPFs arise from anterior compression and posterior tension across the plateau, and often result in concomitant ligamentous damage and significant knee joint instability [2]. Based on the direction of force, HTPFs are classified into pure hyperextension, hyperextension-valgus, hyperextension-varus and hyperextension-bicondylar, with the latter recognised as the most common type [2].

Hyperextension-bicondylar fractures, as seen in our case, comprise of extensive anteromedial metaphyseal comminution and loss of normal posterior sloping of the plateau [2]. These fractures are often unstable, making accurate anatomical restoration difficult [5].

Despite no direct reference to hyperextension-bicondylar non-union in the literature, more complex fracture patterns, often secondary to high-energy trauma, are more likely to disrupt blood supply and surrounding tissues, leading to greater rates of non-union (10–20 %) [5]. Interestingly, similar hyperextension injuries have been observed in obese patients involved in low-velocity trauma through anterior crushing [2].

A literature review identified 13 cases of neglected tibial plateau non-union, of which only one specifically referred to bicondylar involvement [6]. There is no definitive guidance as to when to undertake surgery. Each case is evaluated individually, considering both non-operative and surgical factors; careful pre-operative planning is imperative to minimise intraoperative complications [7]. Non-operative treatment has been suggested when there is minimal displacement, defined as an articular fracture gap and/or step-off of <2 mm [8]. Pean et al., however, have suggested that surgical management is indicated when patients have >2 mm articular incongruence, an open fracture, condylar widening of >5 mm, >5 degrees of varus-valgus instability on examination and minimal/no pre-existing osteoarthritic knee changes; patients must also be medically stable for surgery [9]. Despite this, in a review of previous studies, Giannoudis et al. reported that articular gaps and step-offs ≤10 mm are well-tolerated and have the potential for non-operative management [10].

A number of factors may have contributed to the initial decision not to operate. These included her smoking history, severe obesity and poor mobility. Traditionally, more complex tibial plateau injuries are managed with extensile approaches and plating, as non-operative techniques (traction and casting), have shown inconsistencies in providing support and maintaining reduction [7]. This strategy was used in our case to mobilise the depressed plateau segments, correct malalignment, restore mechanical axis, respect soft tissues, and provide adequate stability until union was achieved. We also used a bone substitute to fill subchondral voids and augment the fixation. We did not encounter any serious complications, in contrast to other reports where authors noted leg length discrepancies (1-2 cm), a superficial wound infection and flexion contracture. This can be attributed to our meticulous pre-operative planning and appropriate handling of the surrounding soft tissue envelope.

In summary, open reduction and internal fixation of neglected bicondylar communited fracture types are difficult procedures, requiring extensive skill and are not without complication risk (e.g., wound dehiscence and infection), regardless of patient factors. Making the decision to not operate on tibial plateau fractures increases non-union likelihood. This risk is generally low, but is significantly greater in those with more complex fractures.

## CRediT authorship contribution statement

**Sophia M Wakefield**: Visualization, Investigation, Writing – Original Draft.

**Vasileios P Giannoudis**: Writing – Reviewing and Editing.

**Peter V Giannoudis**: Supervision, Conceptualization, Writing – Reviewing and Editing.

## Funding statement

This research did not receive any specific grants/funding.

## Consent

Written informed consent was obtained from the patient. The authors confirm the manuscript is sufficiently anonymised in line with the anonymization policy stated in the “Guide for Authors”, and does not contain personal and/or medical information about any identifiable individual.

## Declaration of competing interest

The authors declare that they have no known competing financial interests and/or personal relationships with other people or organisations that could have appeared to influence the work submitted.
